# Clinical Characteristics of Chronic Pancreatitis in a Saudi Arabian Population: A Retrospective Cohort Study at Riyadh Second Health Cluster

**DOI:** 10.1007/s44197-025-00504-9

**Published:** 2026-02-18

**Authors:** Abed Al Lehibi, Omar Hamad Alrashedi, Abrar Humaidi Alhubayshi, Nasser Yousef Al Howaish, Abdullah Hamzah Bawazir, Zainab Ahmed Al Hubail, Hussain Mohammed Al Hassan, Hatem Ayed Alharbi, Zainab Hisham Alabduljabbar, Ahmed Mohammed Almazni, Huda Hassan Alwusaybie

**Affiliations:** 1 Gastroenterology & Hepatology Department in King Fahad Medical City (KFMC), Riyadh, Saudi Arabia; 2 Faculty of Medicine, Dar Al Uloom University, Riyadh, Saudi Arabia; 3 Department of Gastroenterology, King Fahad Medical City, Riyadh, Saudi Arabia; 4 Department of Internal Medicine, King Fahad Medical City, Riyadh, Saudi Arabia; 5 Dar Al Uloom University, Riyadh, Saudi Arabia; 6 Department of Internal Medicine, College of Medicine, Dar Al Uloom University, Riyadh, Saudi Arabia

**Keywords:** Chronic pancreatitis, Non-alcoholic pancreatitis, Risk factors, Complications, Saudi arabia

## Abstract

**Background:**

Chronic pancreatitis (CP) is a progressive inflammatory condition with regional differences in prevalence and etiology. Though alcohol and smoking are primarily the risk factors in Western populations, CP resulted from alcohol is rare in Saudi Arabia due to cultural differences. Our study aim to characterize the Etiology, clinical presentation, and complications of CP in Saudi Arabian adults who are minimally alcohol exposure.

**Methods:**

Our retrospective research highlights on patients who are 14 years of age and older that diagnosed with CP, who are treated at King Fahad Medical City from the timeline of 2013 until 2024. The Demographics, clinical presentations, laboratory, radiological and comorbidity data were obtained from the electronic records of KFMC then analysed by SPSS version 27 program. Our study is approved ethically by the KFMC Research Center, and we ensure all participants’ data remained anonymous.

**Results:**

We identified 117 CP patients (65% female, 95.7% Saudi) with a median age of 42 years and median disease duration of 4 years. Smoking was reported in 40.2% of cases. The predominant symptom was right upper quadrant pain (80.3%), followed by fever (12%), jaundice (11.1%), and pruritus (10.3%). Median laboratory values included amylase 74 U/L, lipase 39.4 U/L, calcium 2.36 mmol/L, triglycerides 1.35 mmol/L, and IgG4 0.847 g/L. Frequent complications included pancreatic leak/fistula (46.2%), pseudocysts (27.4%), bile duct obstruction (26.5%), and pancreatic carcinoma (13.7%). New-onset diabetes and duodenal obstruction each occurred in 9.5%, while metabolic bone disease and autoimmune hepatitis were rare (0.9%).

**Conclusion:**

In Saudi Arabia, CP often occurs without alcohol consumption, but with smoking emerging as a potential risk factor. Despite this non-alcoholic profile, the risk of serious complications, including pancreatic cancer, remains high. Multicentric studies are needed to clarify risk factors and optimize the management.

**Supplementary Information:**

The online version contains supplementary material available at 10.1007/s44197-025-00504-9.

## Introduction

Chronic pancreatitis (CP) is an inflammatory pancreatic disease causing irreversible tissue damage and fibrosis and progressively impairing exocrine and endocrine functions [1, 2]. In the U.S., CP incidence ranges from 5 to 8 per 100,000 person-years, with a prevalence of 42–73 per 100,000 adults [3]. Japan reported a rising number of CP cases, from 32,000 in 1994 to 42,000 in 1999, with incidence increasing from 5.4 to 5.77 per 100,000 [4]. Western populations show a prevalence of ~ 70 per 100,000, with one-third exhibiting exocrine insufficiency [5]. Denmark reported an incidence of 12.6 per 100,000 person-years, higher in men (16.7) than women (8.6) and a prevalence of 153.9 per 100,000 in 2016 [6]. CP is complicated by multiple issues, such as pancreatic pseudocysts caused by ductal obstruction and fibrosis [7, 8]. These cysts may cause pain, structural compression, or infection, often requiring endoscopic drainage [7]. CP also increases vascular risks, including arterial pseudoaneurysms and splanchnic vein thrombosis. These complications can lead to hemorrhage or portal hypertension [9, 10].

Alcohol abuse is the dominant risk factor for CP in Western populations [11]. Excessive alcohol consumption causes toxic-metabolic damage to pancreatic acinar cells, triggering inflammation and fibrosis [11]. Cigarette smoking is another independent risk factor that worsens pancreatic injury and accelerates fibrosis [12, 13]. These environmental factors increase pancreatic toxin exposure, interacting with other predisposing conditions to promote disease progression [11]. Genetic factors also play a role in CP, especially in cases without significant alcohol exposure [14, 15]. Mutations in SPINK1, PRSS1, and CFTR disrupt pancreatic protective mechanisms, increasing susceptibility to CP. SPINK1 mutations are strongly linked to tropical calcific pancreatitis, prevalent in malnourished populations in developing countries [14, 15]. Nutritional deficiencies increase oxidative stress in childhood and further contribute to CP risk in Asia and Latin America [16, 17].

CP presents with persistent abdominal pain, exocrine insufficiency leading to maldigestion, and endocrine dysfunction causing diabetes mellitus [18, 19]. The pain, often chronic and debilitating, significantly impairs the quality of life and is a primary reason for medical intervention [18, 20]. Exocrine insufficiency manifests as steatorrhea, weight loss, and malnutrition, requiring pancreatic enzyme replacement [18, 19]. Endocrine dysfunction results from progressive islet cell loss, leading to diabetes [18, 19]. Laboratory findings in CP include transiently elevated amylase and lipase during acute flares, though these may normalize in advanced disease [21]. Fecal elastase levels below 200 µg/g indicate exocrine insufficiency, with values < 50 µg/g suggesting severe deficiency [22]. Autoimmune pancreatitis (AIP), a CP subtype, often presents with elevated IgG4 (> 135 mg/dL) and autoantibodies [23, 24].

Initial management of CP focuses on no-invasive measures, including alcohol and smoking cessation, dietary modifications, pancreatic enzyme replacement for malabsorption, and analgesics for pain control [25]. However, up to 60% of patients eventually require endoscopic or surgical interventions due to disease progression [25]. Endoscopic retrograde cholangiopancreatography (ERCP) serves as a first-line minimally invasive approach, facilitating pancreatic duct stenting, stricture dilation, stone extraction, and pseudocyst drainage with high success rates [26, 27]. When conservative and endoscopic treatments fail, surgical options such as lateral pancreaticojejunostomy or duodenum-preserving pancreatic head resection offer long-term pain relief and functional improvement [26, 28]. While alcohol consumption is a leading cause of chronic pancreatitis globally, its prevalence is notably low in Saudi Arabia due to strict cultural and legal prohibitions on alcohol. Insofar as the epidemiology, etiology, and clinical characteristics of chronic pancreatitis in Saudi Arabia are concerned, there is a significant gap in the literature. No prior studies have systematically investigated the prevalence, risk factors, or complications of chronic pancreatitis in this population. This study aims to address this gap by examining the probable etiology and presentations of chronic pancreatitis among adult patients in Saudi Arabia.

## Methods

### Study Design

This retrospective cohort study is designed to investigate the prevalence, etiology, and comorbidities of chronic pancreatitis (CP) among adult patients admitted to King Fahad Medical City (KFMC), Riyadh, Saudi Arabia, between January 2013 and January 2024. The retrospective design was chosen to leverage existing medical records, minimize resource-intensive data collection, and ensure robust analysis of historical trends.

### Study Setting and Duration

Study Setting and DurationThe study will be conducted at King Fahad Medical City (KFMC), a tertiary healthcare center in Riyadh, Saudi Arabia. The data collection period spans January 2013 to January 2024, capturing a comprehensive 11-year timeframe to ensure adequate sample size and representation of CP cases.

### Study Population

The study population consists of adult patients (≥ 14 years old) diagnosed with chronic pancreatitis at KFMC during the study period. Patients with incomplete medical records or those diagnosed outside KFMC were excluded.

### Variables

Variables collected from the medical records include demographic data such as age, gender, nationality, marital status, smoking status, and body mass index (BMI); clinical data such as symptoms (fever, right upper quadrant pain, jaundice, pruritus), vital signs at diagnosis (blood pressure, heart rate, respiratory rate, temperature, oxygen saturation), and clinical status (alive or expired); laboratory data such as amylase, lipase, calcium, triglycerides (TG), and IgG-4 levels; radiological findings such as CT, MRCP, ERCP, and endoscopic ultrasound results; and comorbidities such as pancreatic cancer, pancreatic pseudocyst, pancreatic leak or fistula, new-onset diabetes mellitus (DM), splenic vein thrombosis, duodenal obstruction, common bile duct (CBD) obstruction, metabolic bone disease, and autoimmune hepatitis.

### Data Sources and Measurement

Data were extracted from the KFMC electronic medical records system (Epic Research Module). Variables were recorded in an Excel spreadsheet, and patient identifiers were anonymized in compliance with the Helsinki protocol. The dataset included both demographic and clinical variables, as well as laboratory and radiological findings.

### Bias

To minimize bias, all eligible patients were included using predefined criteria, reducing selection bias. Information bias was controlled by systematically extracting data from standardized records.

### Sample Size and Sampling Method

A convenience sampling method was adopted, enrolling all eligible adult patients (≥ 18 years old) diagnosed with chronic pancreatitis at KFMC during the study period.

### Statistical Methods

Data analysis will involve descriptive statistics (medians, quartiles, frequencies) to summarize demographic, clinical, and laboratory variables. All analyses will be performed using SPSS 27.

### Ethical Considerations

The study adheres to the Helsinki protocol to ensure patient confidentiality and privacy. Personal identifiers derived from the Epic system were removed from the dataset before analysis.

### Data Management

Data will be stored securely in password-protected Excel files, with access restricted to the research team. Backups will be maintained to prevent data loss.

## Results

### Baseline Characteristics

A total of 117 patients diagnosed with chronic pancreatitis were included in the study. The demographic characteristics of patients are listed in Table 1. The median age of patients is 42 years old (interquartile range [IQR] 28–56.5). The median age at diagnosis was 36 years old (IQR 22–52.3), and the median duration of the disease was 4 years (IQR 3–6). The majority of patients are female (65%) and Saudi civilians are (95.7%). The median body mass index (BMI) was 25.26 (IQR 20.68–30.36). Regarding smoking status, 40.2% of patients were smokers.

**Table 1:** Baseline characteristics of the study population (*n* = 117)

Variable	Value
Median age, years (IQR)	42 (28–56.5)
Median age at diagnosis, years (IQR)	36 (22–52.3)
Median disease duration, years (IQR)	4 (3–6)
Female, n (%)	76 (65.0)
Saudi nationality, n (%)	112 (95.7)
Median BMI (IQR)	25.26 (20.68–30.36)
Smokers, n (%)	47 (40.2)

### Clinical and Lab Findings

Right upper quadrant (RUQ) pain was the most common symptom, reported in 80.3% of patients. Fever was present in 12% of patients, while jaundice and pruritus were observed in 11.1% and 10.3% of patients, respectively. The median amylase level was 74 U/L (IQR 36.5–192), and the median lipase level was 39.4 U/L (IQR 15.2–306.35). The median calcium level was 2.36 mmol/L (IQR 2.26–2.46), and the median triglyceride (TG) level was 1.35 mmol/L (IQR 0.82–2.02). The median IgG-4 level was 0.847 g/L (IQR 0.352–2.852).

### Complications

Pancreatic pseudocyst was observed in 27.4% of patients, and pancreatic leak or fistula was found in 46.2% of patients. Pancreatic carcinoma was evident in 13.7% of patients, and new-onset diabetes mellitus was reported in 9.5% of patients. Splenic vein thrombosis and duodenal obstruction were each observed in 9.5% of patients, and common bile duct obstruction was observed in 26.5% of patients. Metabolic bone disease and autoimmune hepatitis were rare, each occurring in 0.9% of patients.

## Discussion

Our study analyzed 117 patients with chronic pancreatitis (CP), predominantly female (65%) and Saudi nationals (95.7%). The median age of the cohort was 42 years, with a median age at diagnosis of 36 years and a disease duration of 4 years. Right upper quadrant (RUQ) pain was the most common symptom, reported in 80.3% of patients, while fever, jaundice, and pruritus were less frequent, occurring in 12%, 11.1%, and 10.3% of cases, respectively. Laboratory findings revealed median amylase and lipase levels of 74 U/L and 39.4 U/L, respectively, alongside median calcium and triglyceride levels of 2.36 mmol/L and 1.35 mmol/L. The median IgG-4 level was 0.847 g/L. Complications encompassed pancreatic pseudocyst observed in 27.4% of patients and pancreatic leak or fistula in 46.2%. New-onset diabetes mellitus was reported in 9.5% of patients, whereas splenic vein thrombosis and duodenal obstruction each occurred in 9.5% of cases, and common bile duct obstruction occurred in 26.5% of cases. Metabolic bone disease and autoimmune hepatitis were rare, each affecting 0.9% of patients.

Our study cohort had a smoking prevalence of 40.2%, aligning with reports linking smoking to pancreatic dysfunction [29]. Despite alcohol being a leading global cause of CP, its role in this cohort was likely minimal due to cultural prohibitions in Saudi Arabia [30]. The median triglyceride level was 1.35 mmol/L (IQR 0.82–2.02), ruling out severe hyperlipidemia as a major contributor. IgG-4 levels (median 0.847 g/L, IQR 0.352–2.852) did not suggest an autoimmune origin in most cases. Calcium levels (median 2.36 mmol/L, IQR 2.26–2.46) were within normal range, excluding hypercalcemia as a probable etiology. Other possible mechanisms for CP beyond the effect of smoking comprise genetic mutations such as PRSS1, SPINK1, or CFTR gene variants, which predispose individuals to hereditary CP [31]. In addition, idiopathic CP accounts for a significant proportion of cases (10–40%) in Western populations [30]. Structural abnormalities, such as pancreas divisum or post-traumatic injury (1%), may also contribute [30].

Smoking significantly impairs pancreatic duct cell function, as evidenced by reduced bicarbonate secretion in both current and former smokers compared to never-smokers [29]. The relative risk of duct cell dysfunction was 2.2-fold higher in smokers, independent of age, gender, alcohol intake, or CP diagnosis. Moreover, smoking accelerates disease progression, particularly in late-onset CP, by promoting pancreatic calcifications [32, 33]. A dose-dependent relationship exists, with heavy smoking (≥ 35 pack-years) increasing CP risk by 13-fold among heavy drinkers [34]. This suggests that smoking duration and intensity influence CP development, even in the absence of significant alcohol consumption.

Non-alcoholic CP (NACP) encompasses distinct clinical subtypes with variable natural histories. Early-onset idiopathic CP (IECP) presents in the first two decades with severe pain but slow progression to structural damage, resembling hereditary pancreatitis in its protracted course [32, 33, 35]. In contrast, late-onset idiopathic CP (ILCP), emerging after age 50, usually follows an indolent, painless course with insidious exocrine/endocrine dysfunction [33]. Both forms progress more slowly than alcoholic CP, with delayed calcification and pancreatic insufficiency [32, 35]. Histologically, NACP is characterized by prominent periductal lymphocytic infiltrates (predominantly CD4 + and CD8 + T cells), duct destruction, and disproportionate perilobular fibrosis [36]. Genetic susceptibility —albeit milder than in hereditary CP—also plays a role, with reports of higher frequencies of PRSS1, SPINK1, or CFTR mutations being detected in patients with NACP and potential autoimmune involvement, evidenced by MHC class II expression on ductal epithelium [31, 33, 36]. The long-term prognosis of NACP is more favorable than alcoholic forms with a slower progression and less mortality; nonetheless, subsequent development of pancreatic cancer remains a major concern [30, 31, 35, 37].

Our study offers valuable insights into chronic pancreatitis in a Saudi Arabian population, where alcohol consumption is minimal due to cultural and legal restrictions. Our comprehensive analysis of 117 patients provided detailed demographic, clinical, and laboratory data. This dataset allows a thorough understanding of different presentations and complications in non-alcoholic contexts. However, as a single-center retrospective study, our findings may not be generalizable to the broader population. The absence of detailed alcohol consumption data, despite its low prevalence, could introduce residual confounding. Furthermore, the reliance on existing medical records may have led to incomplete data for certain variables, such as IgG-4 levels, which were only available for a subset of patients. Future research should expand upon these findings through multi-center studies to enhance generalizability and explore the interplay of genetic, environmental, and lifestyle factors in chronic pancreatitis.

## Conclusion

In conclusion, this study details the characteristics of chronic pancreatitis (CP) in a Saudi Arabian population, where alcohol plays a minimal role due to cultural prohibitions. Smoking was prevalent and may have been a significant risk factor associated with pancreatic dysfunction and disease progression. The cohort exhibited a high prevalence of complications, such as pancreatic pseudocysts and leaks. Non-alcoholic CP has distinct clinical and histopathological features, with genetic and autoimmune factors potentially contributing to disease pathogenesis. While the prognosis of non-alcoholic CP appears more favorable than alcoholic forms, long-term risks such as pancreatic cancer remain a concern, as evident from our cohort. Further multi-center studies are needed to validate these findings and explore underlying genetic and environmental interactions.

## Supplementary Information

Below is the link to the electronic supplementary material.

Supplementary Material 1 (DOCX 22.7 KB)

## Data Availability

Data supporting the findings of this study are available from the corresponding author upon reasonable request, in compliance with institutional regulations.
