# Supplementary material for: Clinical Characteristics of Chronic Pancreatitis in a Saudi Arabian Population: A Retrospective Cohort Study at Riyadh Second Health Cluster
Source: J Epidemiol Glob Health. 2026 Feb 18;16(1):17. doi: 10.1007/s44197-025-00504-9 (PMC12923717; doi:10.1007/s44197-025-00504-9)
Supplement: Supplementary file 1 — Supplementary Material 1 (DOCX 22.7 KB) [file 44197_2025_504_MOESM1_ESM.docx]

**Table 1:** Demographic Characteristics of the Included Patients

| **Variables** |  | **N (%)** |
| --- | --- | --- |
| **Age** |  |  |
|  | Median (Q1-Q3) | 42 (28-56.5) |
| **Age at diagnosis (n=110)** |  |  |
|  | Median (Q1-Q3) | 36 (22-52.3) |
| **Duration of disease (n=111)** |  |  |
|  | Median (Q1-Q3) | 4 (3-6) |
| **BMI (n=115)** |  |  |
|  | Median (Q1-Q3) | 25.26 (20.68-30.36) |
| **Sex** |  |  |
|  | Female | 76 (65%) |
|  | Male | 41 (35%) |
| **Nationality** |  |  |
|  | Saudi | 112 (95.7%) |
|  | Non-Saudi | 4 (3.4%) |
|  | Missing | 1 (0.9%) |
| **Marital status** |  |  |
|  | Single | 44 (37.6%) |
|  | Married | 68 (58.1%) |
|  | Missing | 5 (4.3%) |
| **Smoking** |  |  |
|  | Yes | 47 (40.2%) |
|  | No | 67 (57.3%) |
|  | Missing | 3 (2.5%) |

**Table 2:** Clinical Data of the included patients

| **Variables** |  | N (%) |
| --- | --- | --- |
| **Fever** |  |  |
|  | Yes | 14 (12.0%) |
|  | No | 100 (85.4%) |
|  | Missing | 3 (2.6%) |
| **RUQ pain** |  |  |
|  | Yes | 94 (80.3%) |
|  | No | 20 (17.1%) |
|  | Missing | 3 (2.6%) |
| **Jaundice** |  |  |
|  | Yes | 13 (11.1%) |
|  | No | 101 (86.3%) |
|  | Missing | 3 (2.6%) |
| **Pruritis** |  |  |
|  | Yes | 12 (10.3%) |
|  | No | 101 (86.3%) |
|  | Missing | 4 (3.4%) |

**Table 3:** Labs of the included patients

| **Variables** | **Median** | **(Q1 – Q3)** |
| --- | --- | --- |
| **Amylase (n=80)** |  |  |
|  | 74 | (36.5 - 192) |
| **Lipase (n=69)** |  |  |
|  | 39.4 | (15.2 - 306.35) |
| **Calcium (n=113)** |  |  |
|  | 2.36 | (2.26 – 2.46) |
| **Triglycerides (n=79)** |  |  |
|  | 1.35 | (0.82 – 2.02) |
| **IgG-4 (n=32)** |  |  |
|  | 0.847 | (0.352 – 2.852) |

**Table 4:** History of the Complications of the included patients

| **Variables** |  | N (%) |
| --- | --- | --- |
| **Pancreatic Carcinoma** |  |  |
|  | Yes | 16 (13.7%) |
|  | No | 101 (86.3%) |
| **Pancreatic Pseudocyst** |  |  |
|  | Yes | 32 (27.4%) |
|  | No | 85 (72.6%) |
| **Pancreatic leak or Fistula** |  |  |
|  | Yes | 54 (46.2%) |
|  | No | 63 (53.8%) |
| **New Onset DM** |  |  |
|  | Yes | 10 (9.5%) |
|  | No | 107 (91.5%) |
| **Splenic vein thrombosis** |  |  |
|  | Yes | 10 (9.5%) |
|  | No | 107 (91.5%) |
| **Duodenal Obstruction** |  |  |
|  | Yes | 10 (9.5%) |
|  | No | 107 (91.5%) |
| **CBD Obstruction** |  |  |
|  | Yes | 31 (26.5%) |
|  | No | 86 (73.5%) |
| **Metabolic Bone Disease** |  |  |
|  | Yes | 1 (0.9%) |
|  | No | 116 (99.1%) |
| **Autoimmune Hepatitis** |  |  |
|  | Yes | 1 (0.9%) |
|  | No | 116 (99.1%) |
